# Neurologic Manifestations of Long COVID Affect Adult Females More Severely Than Males

**DOI:** 10.1002/acn3.70468

**Published:** 2026-07-21

**Authors:** Hannah Kopinsky, Melissa Lopez, Janet Miller, Millenia Jimenez, Eric M. Liotta, Igor J. Koralnik

**Affiliations:** ^1^ Davee Department of Neurology Northwestern University Feinberg School of Medicine Chicago Illinois USA

**Keywords:** Covid‐19, long Covid, neurology, postacute sequelae of SARS‐CoV‐2 infection, sex differences

## Abstract

**Objective:**

To characterize differences in neurologic manifestations of postacute sequelae of SARS‐CoV‐2 infection (Neuro‐PASC) between females and males.

**Methods:**

Cross‐sectional study of the first consecutive 261 posthospitalization Neuro‐PASC (PNP) and 2068 nonhospitalized Neuro‐PASC (NNP) patients evaluated at the Neuro‐COVID clinic between May 2020 and August 2025. Patients were classified based on biological sex.

**Results:**

There were more females than males in PNP (56.3% vs. 43.7%; *p* = 0.004) and NNP patients (66.3% vs. 33.7%; *p* < 0.0001). An average of 16 months after symptom onset, females from both groups more frequently reported ≥ 4 neurologic symptoms than males (PNP: 79.6% vs. 64.9%; *p* = 0.007. NNP: 76.1% vs. 64.6%; *p* < 0.0001). Both PNP and NNP females reported more frequently numbness/tingling, dizziness, headache and pain than males, while only NNP females reported more frequently brain fog, myalgias, dysgeusia and anosmia than NNP males. In addition, females in both groups more frequently reported fatigue, shortness of breath, chest pain and dysautonomia compared to males, while only NNP females reported more frequent gastrointestinal symptoms than NNP male patients. Compared to males, females from both groups reported worse fatigue on the patient‐reported outcome measure information system (PROMIS) questionnaire, and NNP females had worse results in a task of attention on NIH Toolbox cognitive test.

**Interpretation:**

Females suffer from a higher symptom burden of Neuro‐PASC and had worse subjective quality of life and objective cognitive function. These results highlight the importance of timely diagnosis and targeted interventions in female patients who are often negatively affected by gender bias in medicine.

## 1 Introduction

As of March 2026, close to 780 million cases and 7.1 million deaths have been reported since the beginning of the COVID‐19 pandemic. In the United States, there have been more than 103 million cases and 1.2 million deaths [1], and this number is likely underestimated since most people use rapid diagnostic tests at home that are not reported to the World Health Organization. While severe cases of COVID‐19 pneumonia requiring hospitalization have become rare, many people develop lingering symptoms lasting > 3 months and affecting one or more organ systems, defined as the Long COVID syndrome, also called post‐acute sequelae of SARS‐CoV‐2 infection (PASC) [2]. A household pulse survey carried out by the National Center for Health Statistics estimated that 17.9% of US adults ever experienced Long COVID, and that 5.3% currently experienced it at the last study time point in September 2024 [3]. It has been estimated that the global cumulative incidence of Long COVID amounted to more than 400 million people worldwide, with an annual economic impact of one trillion dollars, equivalent to about 1% of the global economy [4].

Neurologic manifestations of PASC (Neuro‐PASC) are the most frequent cause of consultation at our comprehensive COVID Center [5], and include brain fog, headache, dizziness, myalgia, paresthesia, insomnia, alteration of smell and taste as well as intense fatigue impacting patients' quality of life and ability to work. We and others have shown important differences between posthospitalization Neuro‐PASC (PNP) and nonhospitalized Neuro‐PASC (NNP) patients, indicating that they need to be analyzed separately [6]. Indeed, these two populations differ in their demographics, comorbidities, neurologic symptoms and findings as well as patterns of cognitive dysfunction, suggesting distinct etiologies of Neuro‐PASC. While it has long been known that PASC more frequently affects women than men [7, 8, 9, 10, 11, 12], whether an association exists between sex and Neuro‐PASC has not been determined in detail. The aim of this study is to characterize differences in Neuro‐PASC between females and males in both PNP and NNP patients. We hypothesized that since females are more frequently affected by Neuro‐PASC and less likely to improve than males [13], the Neuro‐PASC symptom burden will be greater in females than males in both patient groups.

## 2 Materials and Methods

### 2.1 Patients

We evaluated a total of 2329 patients, including 261 PNP and 2068 NNP patients who were SARS‐CoV‐2‐positive at the Neuro‐COVID‐19 clinic of Northwestern Memorial Hospital, in Chicago, Illinois, between May 2020 and August 2025. The clinic was listed on the Northwestern website without further advertising. Patients had the option to schedule appointments in person or through a televisit without a physician referral requirement.

Inclusion criteria for this study were the same as previously published [6, 7, 14]. In summary, all patients were required to have a confirmed positive COVID‐19 test by SARS‐CoV‐2 PCR, rapid antigen testing, and/or positive serology for SARS‐CoV‐2 prior to vaccination. Patients were also required to have persistent neurologic symptoms for more than 3 months since a confirmed COVID‐19 episode. This is consistent with the WHO and National Academy of Sciences, Engineering, and Medicine (NASEM) long COVID criteria [2, 15] and is more stringent than the initial National Institutes of Health (NIH) PASC criteria, which only required symptoms lasting > 4 weeks [16]. This study was approved by the Northwestern University IRB (STU00212583). Of note, smaller subsets of patients evaluated in the clinic have been reported previously to compare neurologic manifestations between PNP and NNP patients, age groups as well as those with prevaccination infection and breakthrough infection [6, 7, 14]. None of those inter‐group analyzes were duplicated in the present study, which focuses solely on sex differences and the severity of long COVID within each group of PNP and NNP patients.

### 2.2 Procedures

Clinic visits were held in‐person or over televisit, as previously reported [6, 7, 14]. Briefly, patients from 39 US states were evaluated in televisits. Appointments were for 1 h and all patients were evaluated using a standardized template. Patients answered validated Patient Reported Outcome Measurement Information System (PROMIS) questionnaires [17, 18] about their subjective quality of life prior to their appointment. Patients who were able to visit the clinic in‐person completed an objective evaluation of their cognition with the NIH Toolbox v2.1 as previously reported [19, 20, 21]. Both PROMIS and NIH Toolbox results are expressed as T‐scores, with a score of 50 representing the normative mean/median of the US reference population with a standard deviation of 10.

### 2.3 Statistical Analysis

Patient demographics, comorbidities, and signs and symptoms were summarized as either frequency (with percentage), mean (with standard deviation [SD]) of patients for normally distributed variables or median (with inter‐quartile range [IQR]) for nonnormally distributed variables. Differences in demographics were compared by Mann–Whitney and Fisher's exact test. Comorbidity comparisons between groups used *χ* ^2^ analysis or Fisher's exact test for groups with < 5 patients. When analyzing the signs and symptoms data, a Mann–Whitney test was performed to evaluate months from onset, percent recovery, and number of symptoms. Group differences for specific signs or symptoms were compared by *χ* ^2^ analysis or Fisher's exact test for groups with < 5 patients. Between group PROMIS and Toolbox results were presented as a median with IQR. Mann–Whitney test was performed for comparisons within PROMIS and Toolbox scores. Two‐sided *p* ≤ 0.05 was considered the study wide significance threshold and multiple comparison adjustment of the significance threshold was not implemented for the primary statistical analyzes, an approach justified elsewhere in the literature [22]. However, to identify associations where there may be an added level of confidence, we used the Holm–Bonferroni method to indicate in the manuscript tables those associations that survived the addition of multiple hypothesis test adjustment within each patient cohort (PNP and NNP) and category of variables analyzed (demographics, comorbidities, neurologic symptoms, other symptoms, and neurologic sign). These analyzes were performed in GraphPad Prism version 9.0.0. All data from the study were collected and organized in a REDCap database.

To summarize and visualize the multidimensional symptom profiles of the PNP and NNP cohorts and the relationships between the Neuro‐PASC symptoms, we performed multiple correspondence analysis (MCA) using those symptoms reported as present in ≥ 10% of patients in the overall cohort. MCA results are presented graphically as separate patient and symptom point clouds in two‐dimensional space, defined by the first and second principal component dimensions (the two orthogonal axes with the largest portion of the data inertia, or amount of variation, explained by the component). In the MCA graphs, points further from the origin have greater influence on the component axes; patients plotted in similar locations in space have more similar symptom profiles and symptomatic phenotype than patients plotted at greater distance from each other, and symptom categories that occur more frequently together and share phenotypically similar patients are grouped together. This approach facilitates the appreciation of symptom clusters, or phenotypes, based on how frequently specific symptoms coexist within individuals, rather than analyzing individual symptoms in isolation. MCA was performed using the FactoMineR package in R (R version 4.2.1, Vienna, Austria). We used the Kruskal–Wallis rank sum test to identify if sexes differed by their MCA principal components.

## 3 Results

### 3.1 Patient Demographics and Comorbidities

Of 261 PNP patients, the mean age was 57.4 years, 56.3% were female and 43.7% were male (*p* = 0.004). There was no statistically significant difference in age, race, and ethnicities between female and male PNP patients. Of 2068 NNP patients, the mean age was 47.6 years, 66.3% were female and 33.7% were male (*p* < 0.0001). There was no difference in age and ethnicity between the female and male NNP patients, but there was a statistically significant difference in race (*p* < 0.0001), driven by a higher frequency of Whites in the male group (Table 1).

**TABLE 1:** Demographics comparison between female and male posthospitalization and nonhospitalized Neuro‐PASC patients.

	Overall PNP	Female PNP	Male PNP	*p*	Overall NNP	Female NNP	Male NNP	*p*
Population total, *n* (%)	261	147 (56.3)	114 (43.7)	**0.004** ^a^	2068	1371 (66.3)	697 (33.7)	**< 0.0001** ^a^
Age, years (mean (1SD))	57.4 (13.7)	56.8 (12.7)	58.2 (14.8)	0.1346	47.6 (15.1)	47.6 (14.6)	47.6 (15.9)	0.9034
Race, *n* (%)				0.1663				**< 0.0001** ^a^
White/Caucasian	147 (56.3)	78 (53.1)	69 (60.5)		1486 (71.9)	959 (69.9)	527 (75.6)	
Black or African American	50 (19.2)	33 (22.4)	17 (14.9)		406 (19.6)	118 (8.6)	288 (4.0)	
Asian	7 (2.7)	2 (1.4)	5 (4.4)		66 (3.2)	39 (2.8)	27 (3.9)	
America Indian/Alaskan Native	5 (1.9)	3 (2)	2 (1.8)		5 (0.2)	3 (0.2)	2 (0.3)	
Multiracial	3 (1.1)	3 (2)	0 (0)		17 (0.8)	11 (0.8)	6 (0.9)	
Other	2 (0.8)	2 (1.4)	0 (0)		57 (2.8)	38 (2.8)	19 (2.7)	
Not specified	9 (3.4)	4 (2.7)	5 (4.4)		126 (6.1)	83 (6.1)	43 (6.2)	
Ethnicity, *n* (%)				0.5763				0.1653
Not Hispanic or Latino	209 (80.1)	119 (81)	90 (78.9)		1864	1167 (85.1)	604 (86.7)	
Hispanic or Latino	38 (14.6)	22 (15)	16 (14)		165	120 (8.8)	45 (6.5)	
Not specified	14 (5.4)	6 (4.1)	8 (7)		132	84 (6.1)	48 (6.9)	
Visit type, *n* (%)				0.4271				0.6409
In‐person	174 (66.7)	101 (68.7)	73 (64)		1473	972 (70.9)	501 (71.9)	
Televisit	87 (33.3)	46 (31.3)	41 (36)		595	399 (29.1)	196 (28.1)	
SARS‐CoV‐2 (+), *n* (%)	261 (100)	147 (100)	114 (100)	1	2068 (100)	1371 (100)	697 (100)	1
SARS‐CoV‐2 vaccination, *n* (%)				0.07				0.2207
Yes	222 (85.1)	119 (81)	103 (90.4)		1841 (89)	1214 (88.5)	627 (90)	
No	32 (12.3)	24 (16.3)	8 (7)		179 (8.7)	128 (9.3)	51 (7.3)	
Unknown	7 (2.7)	4 (2.7)	3 (2.6)		48 (2.3)	29 (2.1)	19 (2.7)	

*Note:* Bold: statistically significant at a threshold of 0.05, without threshold adjustment for multiple hypothesis tests.

^a^ Survives additional statistical threshold adjustment for seven hypothesis tests using the Holm–Bonferroni method.

The distribution of comorbid diagnoses varied between females and males in both the PNP and NNP group. Within the PNP group, the most common preexisting comorbidities were hypertension, endocrine disorders (not including Type 2 diabetes), and lung disease. PNP females more frequently reported comorbid headache than PNP males (17.7% vs. 2.6%; *p* = 0.002), as well as neuropsychiatric disease (16.3% vs. 7%; *p* = 0.02). Within the NNP group, the most common preexisting comorbidities were depression/anxiety, endocrine disorders, and lung disease. NNP females more frequently reported comorbid endocrine disorders (24.7% vs. 14.9%; *p* < 0.0001), depression/anxiety (38.5% vs. 30.1%; *p* = 0.0002), headache (23% vs. 10.5%; *p* < 0.0001), neuropsychiatric disease (20.5% vs. 15.6%; *p* = 0.008), autoimmune disease (8% vs. 2%; *p* < 0.0001), gastrointestinal disease (6.4% vs. 3.9%, *p* = 0.017), and dysautonomia (2.8% vs. 1.1%; *p* = 0.018) compared to NNP males. NNP males more frequently reported hypertension (22.1% vs. 16.3%; *p* = 0.001), lung disease (22.7% vs. 17.5%, *p* = 0.005), dyslipidemia (22.7% vs. 14.3%; *p* < 0.0001), cardiovascular disease (11.8% vs. 7.4%; *p* = 0.001), and Type 2 diabetes (8.2% vs. 4.8%; *p* = 0.002) compared to NNP females (Table 2).

**TABLE 2:** Comorbidities comparison between female and male posthospitalization and nonhospitalized Neuro‐PASC patients.

	Overall PNP	Female PNP	Male PNP	*p*	Overall NNP	Female NNP	Male NNP	*p*
*n*	261	147	114		2068	1371	697	
Preexisting comorbidity *n* (%)								
Hypertension	112 (42.9)	61 (41.5)	51 (44.7)	0.60	378 (18.3)	224 (16.3)	154 (22.1)	**0.001** ^a^
Other endocrine disorders	85 (32.6)	51 (34.7)	34 (29.8)	0.40	443 (21.4)	339 (24.7)	104 (14.9)	**< 0.0001** ^a^
Lung disease	79 (30.3)	45 (30.6)	34 (29.8)	0.89	398 (19.2)	240 (17.5)	158 (22.7)	**0.005**
Depression/anxiety	64 (24.5)	42 (28.6)	22 (19.3)	0.08	738 (35.7)	528 (38.5)	210 (30.1)	**0.0002** ^a^
Dyslipidemia	61 (23.4)	30 (20.4)	31 (27.2)	0.20	354 (17.1)	196 (14.3)	158 (22.7)	**< 0.0001** ^a^
Cardiovascular disease	45 (17.2)	24 (21.1)	21 (14.3)	0.66	184 (8.9)	102 (7.4)	82 (11.8)	**0.001** ^a^
Headache	32 (12.3)	26 (17.7)	6 (2.6)	**0.002** ^a^	388 (18.8)	315 (23)	73 (10.5)	**< 0.0001** ^a^
Neuropsychiatric disease	32 (12.3)	24 (16.3)	8 (7)	**0.02**	390 (18.9)	281 (20.5)	109 (15.6)	**0.008**
Cancer	29 (11.1)	15 (10.2)	14 (12.3)	0.60	129 (6.2)	91 (6.6)	38 (5.5)	0.29
Autoimmune disease	24 (9.2)	18 (12.2)	6 (5.3)	0.05	124 (6)	110 (8)	14 (2)	**< 0.0001** ^a^
Insomnia	17 (6.5)	10 (6.8)	7 (6.1)	0.83	216 (10.4)	143 (10.4)	73 (10.5)	0.96
Peripheral vascular disease	16 (6.1)	10 (6.8)	6 (5.3)	0.61	58 (2.8)	40 (2.9)	18 (2.6)	0.66
Chronic kidney disease	11 (4.2)	5 (3.4)	6 (5.3)	0.54	37 (1.8)	19 (1.4)	18 (2.6)	0.05
Gastrointestinal disease	8 (3.1)	7 (4.8)	1 (0.9)	0.14	115 (5.6)	88 (6.4)	27 (3.9)	**0.017**
Type 2 diabetes	7 (2.7)	34 (23.1)	28 (24.6)	0.80	123 (5.9)	66 (4.8)	57 (8.2)	**0.002** ^a^
Traumatic brain injury	7 (2.7)	2 (1.4)	5 (4.4)	0.25	89 (4.3)	56 (4.1)	33 (4.7)	0.49
Cerebrovascular disease	6 (2.3)	3 (2)	3 (2.6)	1	15 (0.7)	11 (0.8)	4 (0.6)	0.79
Neuromuscular disease	6 (2.3)	3 (2)	3 (2.6)	1	18 (0.9)	11 (0.8)	7 (1)	0.64
Dysautonomia	5 (1.9)	2 (1.4)	3 (2.6)	0.66	46 (2.2)	38 (2.8)	8 (1.1)	**0.018**
Organ transplant	0 (0)	0 (0)	0 (0)	1	7 (0.3)	3 (0.2)	4 (0.6)	0.23
Other	22 (8.4)	8 (5.4)	14 (12.3)	**0.05**	334 (16.2)	219 (16)	115 (16.5)	0.76

*Note:* Bold: statistically significant at a threshold of 0.05, without threshold adjustment for multiple hypothesis tests.

^a^ Survives additional statistical threshold adjustment for 21 hypothesis tests using the Holm–Bonferroni method.

### 3.2 Frequency of Symptoms and Signs of Neuro‐PASC in Females Versus Males

The mean amount of time from onset of symptoms to clinic visit was 15.9 months in the PNP group with no significant difference in time to evaluation between PNP females and PNP males. The mean amount of time from onset of symptoms to clinic visit was 16.8 months in the NNP group with no significant difference in time to evaluation between NNP females and NNP males. The median number of neurologic symptoms attributed to PASC differed between PNP females and males (6 vs. 5; *p* = 0.004) and between NNP females and males (5 vs. 4; *p* < 0.0001).

The 10 most common neurologic symptoms reported by PNP patients include brain fog (88.5%), numbness/tingling (59%), dizziness (56.7%), myalgia (55.2%), headache (54.4%), pain other than chest (51.3%), dysgeusia (41.8%), anosmia (38.3%), tinnitus (37.2%), and blurred vision (33%). PNP females more frequently reported numbness/tingling (66% vs. 50%; *p* = 0.04), dizziness (62.6% vs. 49.1%; *p* = 0.02), headache (59.9% vs. 47.4%; *p* = 0.04), and pain other than chest (57.1% vs. 43.9%; *p* = 0.03) compared to PNP males. PNP females more frequently had ≥ 4 neurologic symptoms (79.6% vs. 64.9%; *p* = 0.007) compared to PNP males. There were no statistically significant neurologic symptoms that were reported more frequently in PNP males compared to PNP females. Other common nonneurologic symptoms reported in the PNP group include fatigue (88.1%), depression/anxiety (69.3%), shortness of breath (68.2%), insomnia (62.5%), chest pain (39.1%), dysautonomia (34.1%), and GI symptoms (25.3%). Of the non‐neurologic symptoms, PNP females more frequently reported fatigue (92.5% vs. 82.5%; *p* = 0.01), shortness of breath (73.5% vs. 61.4%; *p* = 0.03), chest pain (44.9% vs. 31.6%; *p* = 0.02), and dysautonomia (42.2% vs. 23.7%; *p* = 0.001) compared to PNP males. There were no non‐neurologic symptoms that were reported more frequently in PNP males compared to PNP females.

The 10 most common neurologic symptoms reported by the NNP group include brain fog (86.4%), headache (67.1%), dizziness (59.1%), myalgia (52.9%), numbness/tingling (47.3%), pain other than chest (46.3%), tinnitus (41.3%), anosmia (37.2%), dysgeusia (35.7%), and blurred vision (34.9%). NNP females more frequently reported brain fog (88% vs. 83.2%; *p* = 0.002), headache (70.4% vs. 60.5%; *p* < 0.0001), dizziness (62.4% vs. 52.7%; *p* < 0.0001), myalgia (57.1% vs. 38.7%; *p* < 0.0001), pain other than chest (50% vs. 39%; *p* < 0.0001), numbness/tingling (49.7% vs. 42.8%; *p* = 0.002), anosmia (39.4% vs. 33%; *p* = 0.005), and dysgeusia (38.5% vs. 30.3%; *p* = 0.0002) compared to NNP males. NNP females more frequently had ≥ 4 neurologic symptoms (76.1% vs. 64.6%; *p* < 0.0001) compared to NNP males. There were no statistically significant neurologic symptoms that were reported more frequently in NNP males compared to NNP females. Other common nonneurologic symptoms reported in the NNP group include fatigue (90.6%), depression/anxiety (74.7%), insomnia (59.5%), shortness of breath (47.3%), dysautonomia (37.8%), GI symptoms (35.9%), and chest pain (30.8%). Of the nonneurologic symptoms, NNP females more frequently reported fatigue (91.6% vs. 88.7%; *p* = 0.02), shortness of breath (50% vs. 42.3%; *p* = 0.0006), dysautonomia (41.1% vs. 31.4%; *p* < 0.0001), GI symptoms (39.6% vs. 28.8%; *p* < 0.0001), and chest pain (32.3% vs. 27.7%; *p* = 0.03) compared to NNP males. There were no nonneurologic symptoms that were reported more frequently in NNP males compared to NNP females.

We performed a complete neurologic exam on the 1647 patients who came in‐person to the clinic and a limited exam on the 682 patients who were evaluated via televisit. In the PNP group, 47.1% of patients had an abnormal neurologic exam, and the most frequent findings included short‐term memory deficits in 31.8%, sensory dysfunction in 17.6%, gait dysfunction in 16.1%, deficit in attention in 14.9%, and motor dysfunction in 10.3%. There were no statistically significant differences in neurologic signs on evaluation between PNP females and PNP males. In the NNP group, 45.4% of patients had an abnormal neurologic exam, and the most frequent findings included short‐term memory deficits in 31.2%, deficit in attention in 12.4%, and sensory dysfunction in 11.5%. Females in the NNP group more frequently had an abnormal neurologic exam (46% vs. 44.2%; *p* < 0.0001) and deficit in attention (14.5% vs. 8.3%; *p* < 0.0001) compared to males. Conversely, males in the NNP group more frequently had sensory dysfunction (15.2% vs. 9.6%; *p* < 0.0001) and a movement disorder (1.4% vs. 0.6%; *p* = 0.04) compared to females (Table 3).

**TABLE 3:** Neurologic symptoms and signs comparison between female and male posthospitalization and nonhospitalized Neuro‐PASC patients.

	Overall PNP	Female PNP	Male PNP	*p*	Overall NNP	Female NNP	Male NNP	*p*
*n*	261	147	114		2068	1371	697	
Time from symptom onset to clinic visit (month, mean (1 SD))	15.9 (12.1)	15.8 (12.5)	15.9 (11.7)	0.71	16.8 (12.9)	16.4 (12.8)	17.4 (13.1)	0.06
Neurologic manifestations or symptoms attributed to PASC (median [IQR])	5 [3–7]	6 [4–7]	5 [3–7]	**0.004**	5 [3–7]	5 [4–7]	4 [3–6]	**< 0.0001**
*n* (%)								
≥ 4	191 (73.2)	117 (79.6)	74 (64.9)	**0.007**	1493 (72.2)	1043 (76.1)	450 (64.6)	**< 0.0001** ^a^
Brain fog	231 (88.5)	133 (90.5)	98 (86)	0.25	1786 (86.4)	1206 (88)	580 (83.2)	**0.002** ^a^
Numbness/tingling	154 (59)	97 (66)	57 (50)	**0.04**	979 (47.3)	681 (49.7)	298 (42.8)	**0.002** ^a^
Dizziness	148 (56.7)	92 (62.6)	56 (49.1)	**0.02**	1223 (59.1)	856 (62.4)	367 (52.7)	**< 0.0001** ^a^
Myalgia	144 (55.2)	88 (59.9)	56 (49.1)	0.08	1093 (52.9)	783 (57.1)	310 (38.7)	**< 0.0001** ^a^
Headache	142 (54.4)	88 (59.9)	54 (47.4)	**0.04**	1387 (67.1)	956 (70.4)	422 (60.5)	**< 0.0001** ^a^
Pain other than chest	134 (51.3)	84 (57.1)	50 (43.9)	**0.03**	957 (46.3)	685 (50)	272 (39)	**< 0.0001** ^a^
Dysgeusia	109 (41.8)	68 (46.2)	41 (36)	0.09	739 (35.7)	528 (38.5)	211 (30.3)	**0.0002** ^a^
Anosmia	100 (38.3)	59 (40.1)	41 (36)	0.49	770 (37.2)	540 (39.4)	230 (33)	**0.005**
Tinnitus	97 (37.2)	55 (37.4)	42 (36.8)	0.92	854 (41.3)	563 (41.1)	291 (41.8)	0.76
Blurred vision	86 (33)	53 (36.1)	33 (29)	0.22	723 (34.9)	494 (36)	229 (32.9)	0.15
Ischemic stroke	6 (2.3)	2 (1.4)	4 (3.5)	0.25	27 (1.3)	18 (1.3)	9 (1.3)	0.96
Seizure	4 (1.5)	2 (1.4)	2 (1.8)	0.79	39 (1.9)	28 (2)	11 (1.6)	0.46
Movement disorder	3 (1.1)	1 (0.7)	2 (1.8)	0.41	10 (0.5)	8 (0.6)	2 (0.3)	0.51
Meningitis	1 (0.4)	0 (0)	1 (0.9)	0.25	3 (0.1)	2 (0.1)	1 (0.1)	1
Focal sensory deficit	1 (0.4)	1 (0.7)	0 (0)	0.37	3 (0.1)	2 (0.1)	1 (0.1)	1
Focal motor deficit	0 (0)	0 (0)	0 (0)	1	1 (0.05)	1 (0.07)	0 (0)	1
Encephalitis	0 (0)	0 (0)	0 (0)	1	5 (0.2)	2 (0.1)	3 (0.4)	0.34
Hemorrhagic stroke	0 (0)	0 (0)	0 (0)	1	1 (0.05)	1 (0.07)	0 (0)	1
Polyradiculitis	0 (0)	0 (0)	0 (0)	1	4 (0.2)	2 (0.1)	2 (0.3)	0.60
Other symptoms, *n* (%)								
Fatigue	230 (88.1)	136 (92.5)	94 (82.5)	**0.01**	1874 (90.6)	1256 (91.6)	618 (88.7)	**0.02**
Depression/anxiety	181 (69.3)	106 (72.1)	75 (65.8)	0.27	1544 (74.7)	1040 (75.9)	504 (72.3)	0.07
Shortness of breath	178 (68.2)	108 (73.5)	70 (61.4)	**0.03**	979 (47.3)	686 (50)	293 (42.3)	**0.0006** ^b^
Insomnia	163 (62.5)	93 (63.3)	70 (61.4)	0.75	1231 (59.5)	835 (60.9)	396 (56.8)	0.07
Chest pain	102 (39.1)	66 (44.9)	36 (31.6)	**0.02**	636 (30.8)	443 (32.3)	193 (27.7)	**0.03**
Dysautonomia	89 (34.1)	62 (42.2)	27 (23.7)	**0.001** ^b^	782 (37.8)	563 (41.1)	219 (31.4)	**< 0.0001** ^b^
GI symptoms	66 (25.3)	41 (27.9)	25 (21.9)	0.27	744 (35.9)	543 (39.6)	201 (28.8)	**< 0.0001** ^b^
Sign *n* tested/total (%)								
Abnormal exam	123 (47.1)	68 (46.2)	55 (48.2)	0.74	938 (45.4)	630 (46)	308 (44.2)	**< 0.0001** ^c^
Short‐term memory deficits	83 (31.8)	48 (32.7)	35 (30.7)	0.73	646 (31.2)	435 (31.7)	211 (30.3)	0.49
Sensory dysfunction	46 (17.6)	25 (17)	21 (18.4)	0.76	237 (11.5)	131 (9.6)	106 (15.2)	**0.0001** ^c^
Gait dysfunction	42 (16.1)	25 (17)	17 (14.9)	0.64	176 (8.5)	126 (9.2)	50 (7.2)	0.12
Attention deficit	39 (14.9)	24 (16.3)	15 (13.2)	0.47	257 (12.4)	199 (14.5)	58 (8.3)	**< 0.0001** ^c^
Motor dysfunction	27 (10.3)	17 (11.6)	10 (8.8)	0.46	91 (4.4)	62 (4.5)	29 (4.2)	0.70
Cranial nerve dysf—	14 (5.4)	10 (6.8)	4 (3.5)	0.24	78 (3.8)	52 (3.8)	26 (3.7)	0.94
Cerebellar dysf—	13 (5)	9 (6.1)	4 (14.9)	0.33	69 (3.3)	48 (3.5)	21 (3)	0.55
Movement disorder	2 (0.8)	2 (1.4)	0 (0)	0.21	18 (0.9)	8 (0.6)	10 (1.4)	**0.04**

*Note:* Bold: statistically significant at a threshold of 0.05, without threshold adjustment for multiple hypothesis tests.

^a^ Survives additional statistical threshold adjustment for 20 hypothesis tests using the Holm–Bonferroni method.

^b^ Survives additional statistical threshold adjustment for seven hypothesis tests using the Holm–Bonferroni method.

^c^ Survives additional statistical threshold adjustment for nine hypothesis tests using the Holm–Bonferroni method.

### 3.3 Quality of Life Surveys and Objective Cognitive Tests

**FIGURE 1:**
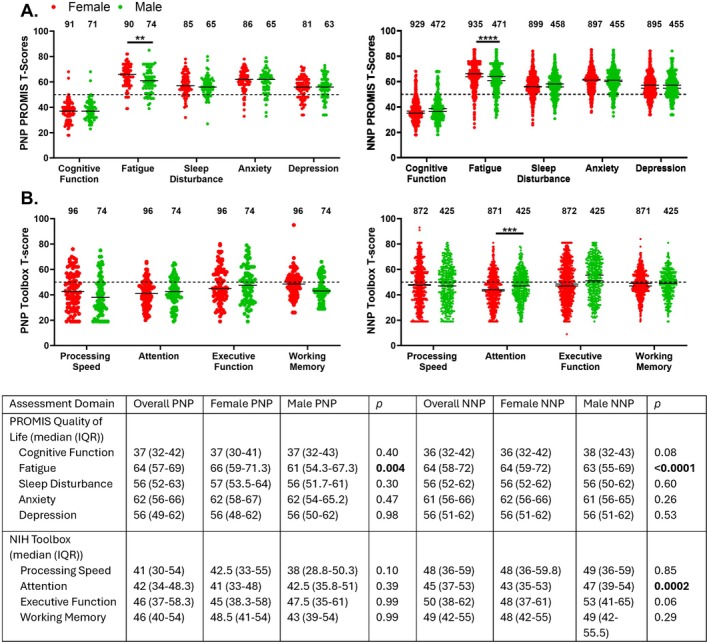
Quality of life measures and cognitive function of PNP and NNP in female and male patients: (A) PROMIS T‐scores for female and male patients in the PNP and NNP groups. (B) NIH toolbox T‐scores for female and male patients in the PNP and NNP groups. PROMIS and NIH toolbox scores were compared between groups using the Mann–Whitney U‐test and two‐sided *p* ≤ 0.05 was considered statistically significant. When comparing quality of life measures, there was a statistically significant difference in fatigue experienced between females and males in both the PNP group (*p* = 0.004) and the NPP group (*p* < 0.0001). When comparing cognitive function, there was no difference between females and males in the PNP group, but females had significantly worse results in the measure of attention compared to males in the NPP group (*p* = 0.0002).

The results of the PROMIS quality‐of‐life surveys and NIH toolbox cognitive tests are shown in Figure 1. Within the PNP and the NNP groups, female patients reported a significantly higher subjective impression of fatigue when compared to male patients (*p* = 0.004 and *p* < 0.0001, respectively). There were no differences between female and male patients in both the PNP and NPP groups for subjective concerns with cognitive function, sleep disturbance, anxiety, or depression (Figure 1A). Within the PNP group, there was no significant difference in objective cognitive function when comparing PNP female and PNP male patient scores on the NIH toolbox. However, within the NNP group, female patients performed significantly worse in the domain of attention compared to male patients (*p* = 0.0002) (Figure 1B).

### 3.4 Multiple Correspondence Analysis

**FIGURE 2:**
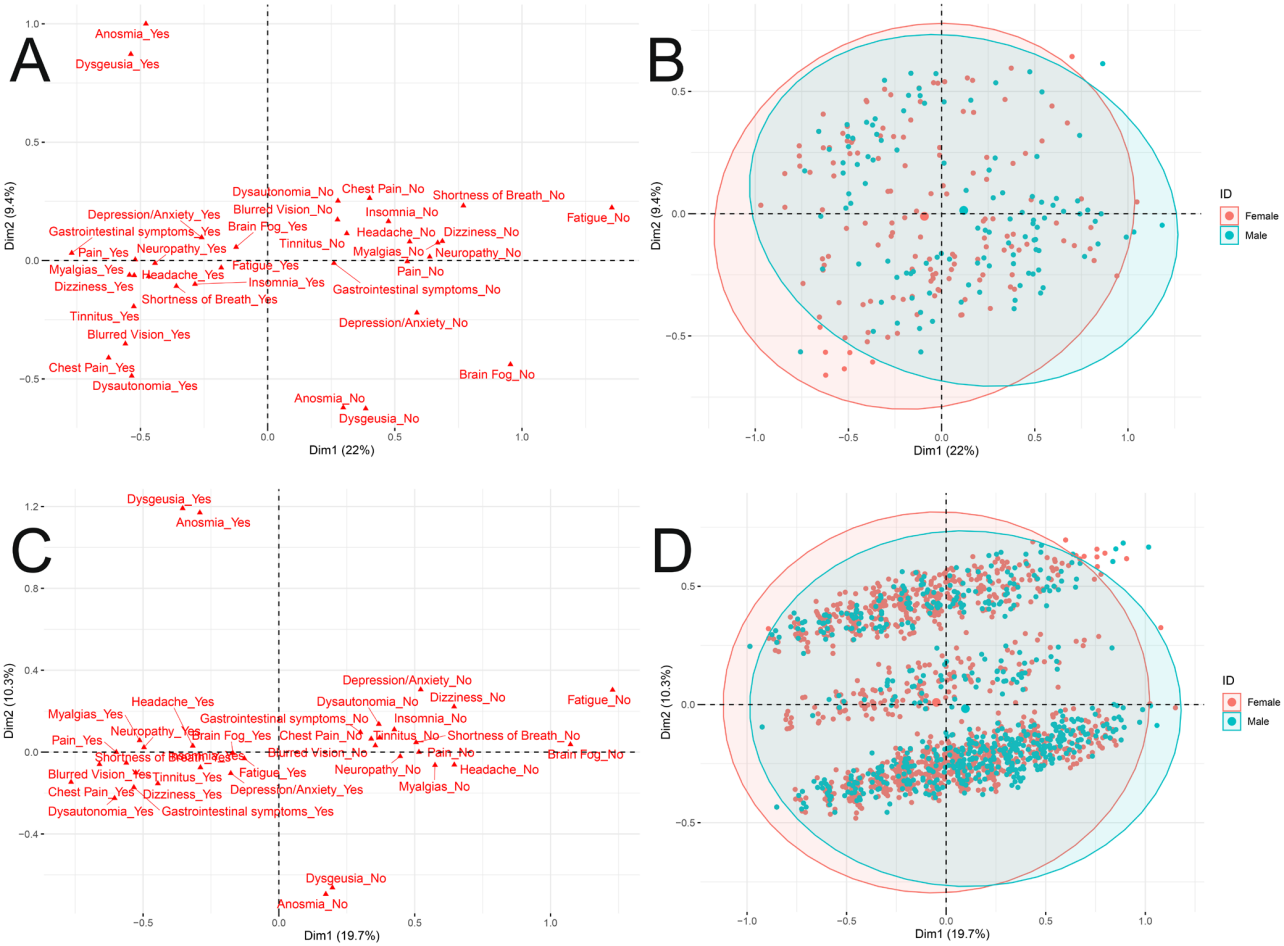
Multiple Correspondence Analysis of Neuro‐PASC symptoms by sex. (A) PNP cohort symptom point cloud. (B) PNP cohort patient point cloud with concentration ellipses by sex. (C) NNP cohort symptom point cloud. (D) NNP cohort patient point cloud with concentration ellipses by sex. Larger‐sized points represent the mean values of each respective sex's distribution. Increasing distance between the origin and a given symptom category indicates a greater contribution of that category to the pole of the corresponding dimension. Symptom categories with similar profiles of patients and that more frequency coexist are grouped closer together. For both PNP and NNP, Dimension 1 globally separates “yes” from “no” symptom categories along a “General Somatic and Neurologic” symptom burden phenotype such that “yes” symptom categories are toward the left of the graph. For example, symptoms such as pain, myalgias, and dizziness cluster closely together on the left, indicating that they frequently coexist. The significant shift of the female patients toward the left reflects clustering toward a high burden of multiple coexisting symptoms (*p* < 0.001). For both PNP and NNP, the presence or absence of anosmia and dysgeusia are the predominant contributors to Dimension 2 and form an axis of Chemosensory loss. The vertical stratification (particularly NNP) along this dimension indicates that the presence of anosmia and dysgeusia is a predominant differentiator of patient subgroups, independent of the General Somatic and Neurologic symptom burden captured by Dimension 1. This dimension of Chemosensory loss also did not differ between the sexes (PNP *p* = 0.51, NNP *p* = 0.63).

Seventeen symptoms were reported as present in ≥ 10% of patients and were therefore included in the MCA, graphically displayed in Figure 2. Interpretation of the MCA graphs in PNP and NNP patients is described in the legend to Figure 2. For the PNP group, Dimension 1 explained 22% of the variance and represents a “General Somatic and Neurologic” phenotype characterized by high correlations (*r* ^2^) with myalgias (0.36), dizziness (0.36), pain (0.28), neuropathy (0.28), shortness of breath (0.28), headache (0.26), chest pain (0.25), and fatigue (0.25); all other symptoms had more auxiliary contributions with *r* ^2^ values between 0.12 and 0.21. Patients on the left of Dimension 1 reported greater coexistence of these “General Somatic and Neurologic” symptoms and generally appear more phenotypically severe. In contrast, Dimension 2 (variance 9.4%) defined a “Chemosensory” phenotype almost exclusively dominated by anosmia (*r* ^2^ = 0.62) and dysgeusia (*r* ^2^ = 0.55), with negligible contribution from other symptoms (all other symptoms having *r* ^2^ values below 0.12). MCA results for PNP patients are displayed in Figure 2A,B.

For the NNP group, Dimension 1 explained 19.7% of the variance and also represented a “General Somatic and Neurologic” phenotype characterized by high correlations (*r* ^2^) with pain (0.31), myalgias (0.29), dizziness (0.29), shortness of breath (0.28), and chest pain (0.26); all other symptoms had more auxiliary contributions with *r* ^2^ values between 0.05 and 0.23. Patients on the left of Dimension 1 reported greater coexistence of these “General Somatic and Neurologic” symptoms and generally appear more phenotypically severe. In contrast, Dimension 2 (variance 10.3%) defined a “Chemosensory” phenotype almost exclusively dominated by anosmia (*r* ^2^ = 0.81) and dysgeusia (*r* ^2^ = 0.79), with negligible contribution from other symptoms (all other symptoms having *r* ^2^ values below 0.04). MCA results for NNP patients are displayed in Figure 2C,D.

The strength of anosmia and dysgeusia dominance in Dimension 2, the “Chemosensory” dimension, creates a visual vertical stratification in the patient point clouds (Figure 2B,D) that identifies the presence of distinct phenotypes based on Chemosensory symptoms that are largely distinct from the other reported symptoms that are represented along the horizontal. The patients in the upper “band” of the graphs have both taste and smell loss, those in the middle “band” have mixed either taste or smell loss, and those in the bottom “band” have neither taste nor smell loss. Patients in all three bands span the spectrum of “General Somatic and Neurologic” symptoms along the horizontal (Dimension 1).

For both PNP (median [IQR]: female −0.114 [−0.479, 0.227] and male 0.151 [−0.276, 0.496]) and NNP (median [IQR]: female −0.059 [−0.360, 0.258] and male 0.130 [−0.198, 0.411]), there was a significant difference in the Dimension 1 “General Somatic and Neurologic” symptom phenotype between sexes (*p* < 0.001). Female patients were more significantly shifted toward the left “yes” pole of this dimension, indicating a phenotype with higher overall burden of symptoms compared to males. No sex differences were observed for the Dimension 2 “Chemosensory” phenotype (PNP *p* = 0.51, NNP *p* = 0.63), suggesting that overall Chemosensory symptoms are affected equally in the sexes.

## 4 Discussion

We and others have previously shown the importance of analyzing data from PNP and NNP patients separately [6, 23, 24, 25]. Our previous studies examined features of Neuro‐PASC according to age over the adult lifespan, and the influence of vaccination on Neuro‐PASC [7, 14, 26]. This study addresses the knowledge gap of the impact of sex on Neuro‐PASC in both PNP and NNP groups.

While the predominance of females presenting with PASC has been widely documented since the beginning of the pandemic [7, 8, 9, 10, 11], our study is novel in that it focuses specifically on Neuro‐PASC. Our data show a higher frequency of females over males in the population of the Neuro‐COVID‐19 clinic, in both the PNP and NNP groups. Females reported a higher prevalence of certain comorbidities and a higher burden of many Neurologic and Non‐Neurologic symptoms of PASC. Females also had significantly worse subjective QoL in the domain of fatigue in both PNP and NNP groups and lower results on objective cognitive tests of attention in the NNP group. By utilizing MCA, we moved beyond the consideration of individual symptom prevalence to visualize the symptom constellation of Neuro‐PASC, the distribution of patients across the phenotypic spectrum, and the degree of coexistence between given symptoms. The MCA analysis supports a sex‐based difference in General Somatic and Neurologic symptoms, with females clustering more densely toward a high burden of symptom coexistence. In contrast, the MCA Dimension 2 was dominated by Chemosensory symptoms, but those Chemosensory symptoms are similarly expressed across both sexes and along the entire spectrum of nonchemosensory General Somatic and Neurologic symptom phenotypes (Dimension 1). This suggests that while General Somatic and Neurologic symptoms are sex‐modulated, the mechanism underlying chemosensory loss may be independent of sex and decoupled from the other Neuro‐PASC symptoms. Indeed, persistent inflammation was observed in the olfactory epithelium of patients with post‐COVID anosmia, which was associated with epithelial damage and neuronal loss even after viral clearance [27].

The prevalence of comorbidities may explain some of the difference in PASC symptoms between females and males. Indeed, patient comorbidities varied by sex in both groups, with co‐morbid headache and neuropsychiatric diseases being more prevalent in females in both PNP and NNP groups. Additionally, female patients in the NNP group more frequently reported comorbid endocrine disorders, depression/anxiety, autoimmune disease, GI disease, and dysautonomia. These differences likely contributed to the findings of more frequently reported Neuro‐PASC symptoms of headache and dysautonomia in female patients of both PNP and NNP groups.

However, comorbidities alone cannot explain the significantly higher burden of PASC symptoms in female participants. Compared to PNP males, PNP females reported significantly greater occurrence of 4/10 most frequent Neurologic and 4/7 non‐Neurologic symptoms of PASC, while compared to NNP males, NNP females reported significantly greater occurrence of 8/10 most frequent Neurologic and 5/7 non‐Neurologic symptoms of PASC. Conversely, not a single Neurologic or non‐Neurologic PASC symptom was reported more frequently in males of both the PNP and NNP groups. These results indicate that females are more broadly affected than males by both Neurologic and non‐Neurologic symptoms of PASC.

These findings also explain that females in both PNP and NNP groups reported significantly worse quality of life than males in the domain of fatigue, and a trend for worse cognition in females of the NNP group. Of note, our data confirm previous studies showing alterations in the moderate range in subjective domains of cognitive function, fatigue, and anxiety, and in the mild range for sleep disturbance and depression in our entire cohort relative to the general population.

Despite the higher burden of neurologic symptoms in PNP females, there was no significant difference on neurologic examination between PNP females and PNP males. However, females in the NNP group more frequently had deficits in attention on the neurologic exam compared to NNP males, which is consistent with the results of the NIH toolbox cognitive test. Males in the NNP group more frequently had sensory dysfunction and abnormal motor exam compared to NNP females, which may be in part due to increased prevalence of co‐morbid vascular risk factors and Type 2 Diabetes observed in NNP males.

### 4.1 Why Are Females More Frequently and Severely Affected by Neuro‐PASC Than Males?

Several studies demonstrate the impact of sex hormones on COVID‐19 infection severity as well as on the development of PASC [28, 29, 30, 31]. PASC is likely due to multiple factors. These may include endothelial dysfunction and alteration of microvascular perfusion leading to mitochondrial impairment, viral persistence triggering a continuous immune response, and immune dysregulation with resultant chronic inflammation and autoimmunity [32, 33, 34]. The hypothesis that the higher prevalence of Neuro‐PASC in young female patients can be largely attributed to immune dysregulation and autoimmunity is supported by several studies. Females have increased adaptive and innate immune responses to acute infections. While it decreases their risk of mortality following acute COVID‐19, it also increases their risk of developing Neuro‐PASC [28, 35, 36, 37]. Additionally, females with PASC had higher expression of the *XIST* gene, which results in an RNA product that is involved in gene modulation on the X chromosome in females but can also act as an auto‐antigen and has been associated with several autoimmune conditions such as rheumatoid arthritis, lupus, and systemic sclerosis that are more frequent in females [29, 38].

Our observations are in‐line with prior studies that revealed Neuro‐PASC to be more prevalent in females compared to male patients [8, 9, 10, 11, 12]. Our study is unique in that it is a single‐center observational study being conducted at a large urban center in the United States with a focus on evaluating Neuro‐PASC in patients from both PNP and NNP groups.

### 4.2 Limitations

Our study has several limitations. Our data derives from the experience of a single center and may be subject to referral bias. However, we evaluated patients both in person and in televisits coming from 39 different US states. Therefore, our population is representative of individuals who seek care at a specialized Long COVID clinic in the US. In addition, to facilitate access to care, we did not request physician referral. About a third of our patients were evaluated in televisit and had a limited neurologic exam compared to those who presented in person. We have previously demonstrated by principal component analyzes that these two populations were largely identical [6]. Due to limitations on human subjects' research during the pandemic, we were unable to test our own control group. However, we used the NIH Toolbox and PROMIS scores, which both reference a US normative population and have been extensively validated for neurologic research. We did not adjust our study wide significance threshold for multiple comparisons in our primary statistical analyzes, since those adjustments may increase the Type II error for those associations that are nonnull [22, 39] and due to the exploratory nature of this study. However, we did indicate associations that withstand a Holm–Bonferroni multiple comparison approach to indicate those associations where there may be an added level of confidence. We also did not assess the effect of vaccination on sex differences in Neuro‐PASC, since the impact and timing of vaccination on Neuro‐PASC was reported in a previous study [14]. Finally, we were not able to evaluate the possible sex‐related influence of SARS‐CoV‐2 variants on Neuro‐PASC, since we could not retrospectively confirm the viral strain for each patient, given this testing was not performed at our institution.

## 5 Conclusion

These data confirm our initial hypothesis that the Neuro‐PASC burden is greater in females than males. While the acute phase of the COVID pandemic may be forgotten by some, Long COVID continues to impact the health and quality of life of many survivors, representing an important public health concern. As memory of the pandemic fades, dismissal and minimization of symptoms, with a risk of misdiagnosis or delay in diagnosis, may unfortunately predominantly affect females. Indeed, young and middle‐aged females are populations that have already suffered from gender inequity in health care recognition [40, 41]. Consequently, this situation may cause a reinforcement of gender bias in medicine.

There is a continued need for research on the root causes of Long COVID and to delineate biomarkers of sex differences in Neuro‐PASC, which will be necessary to devise personalized therapeutic interventions. Knowledge gained from Long COVID may in turn inform etiologies of other chronic conditions that have been associated with infections such as myalgic encephalomyelitis/chronic fatigue syndrome (ME/CFS), which also more severely affect females [42].

## Author Contributions

M.L., J.M., M.J., E.M.L., and I.J.K. contributed to the conception and the design of the study. H.K., M.L., J.M., M.J., E.M.L., and I.J.K. contributed to the acquisition and analysis of the data. H.K., M.L., J.M., E.M.L., and I.J.K. contributed to drafting the text or contributed to preparing the figures.

## Funding

This work was supported in part by the National Institute on Aging of the National Institutes of Health (grant K23AG078705, E.M.L.).

## Conflicts of Interest

The authors declare no conflicts of interest.

## Data Availability

Deidentified data will be made available upon reasonable request by qualified investigators in accordance with institutional policies and applicable ethical regulations.
